# Trajectories of general and central obesity beyond middle age in relation to late‐life cognitive decline and dementia

**DOI:** 10.1002/oby.24208

**Published:** 2025-01-28

**Authors:** Zhengting Liang, Huibo Qin, Binbin Su, Yanping Bao, Michael V. Vitiello, Gang Hu, Yunhe Wang

**Affiliations:** ^1^ School of Traditional Chinese Medicine Xinjiang Medical University Urumqi China; ^2^ Quality Control Department of Liaocheng People's Hospital Shandong China; ^3^ School of Population Medicine and Public Health Chinese Academy of Medical Sciences/Peking Union Medical College Beijing China; ^4^ National Institute on Drug Dependence Peking University Beijing China; ^5^ School of Public Health Peking University Beijing China; ^6^ Department of Psychiatry and Behavioral Sciences University of Washington Seattle Washington USA; ^7^ School of Health Management Xinjiang Medical University Urumqi China; ^8^ Health Management Center of the First Affiliated Hospital of Xinjiang Medical University Urumqi China; ^9^ Nuffield Department of Population Health University of Oxford Oxford UK

## Abstract

**Objective:**

The objective was to evaluate the longitudinal patterns of central and general obesity, identify their genetic and behavioral risk determinants, and investigate the association of distinct obesity trajectories beyond middle age with subsequent cognitive decline and the risk of developing dementia in late life.

**Methods:**

Using a nationally representative, longitudinal, community‐based cohort, we examined trajectory patterns of obesity over a 14‐year span beyond middle age employing latent mixture modeling. We then evaluated their relationship with subsequent cognitive decline through linear mixed models and with the risk of developing dementia using Cox models, adjusting for confounding variables.

**Results:**

Among the 4751 eligible participants (mean age, 58.7 [SD 8.1] years; 57% female), our analysis identified five distinct BMI trajectories and four WC trajectories spanning a 14‐year period. In comparison with individuals in the low‐stable BMI group, characterized by a consistent and healthy body weight (range, 22.8–22.9 kg/m^2^), those in the high‐stable group, maintaining a stable obesity status (range, 34.3–35.4 kg/m^2^), exhibited an elevated risk of developing dementia (odds ratio [OR], 1.43; 95% CI: 1.02 to 2.00) and experienced a more accelerated cognitive decline over 6 years (difference in 6‐year decline, −0.11 SD [95% CI: −0.18 to −0.03]). Similarly, when compared with participants in the low‐stable WC group, indicating a stable and healthy WC (range, 76–79 cm), those in the high‐increasing WC group, showing an increasing trend (range, 115–122 cm), demonstrated an increased risk of developing dementia (OR, 1.57, 95% CI: 1.01 to 2.49) and experienced a swifter cognitive decline (OR: −0.18 [95% CI: −0.28 to −0.07]).

**Conclusions:**

General and central obesity trajectories beyond midlife with persistently high or increasing patterns were significantly associated with an increased risk of developing cognitive decline and dementia in late life. Longitudinal obesity patterns may assist in precise identification of older adults at risk of developing cognitive impairment for targeted intervention.

**Study Importance**

**What is already known?**

- Single measures of adiposity markers such as BMI and waist circumference (WC) are associated with adverse neurocognitive outcomes; however, the association between longitudinal patterns of adiposity trajectories in midlife and cognitive decline and dementia in late life remains unclear.

**What does this study add?**

- Five discrete BMI and four WC trajectory patterns were identified over 14 years, which were associated with genetic factors in a dose–response manner and with behavioral lifestyle factors.
- General and central adiposity trajectories in midlife were associated with subsequent cognitive decline and the risk of developing dementia in late life.

**How might these results affect the direction of research or the focus of clinical practice?**

- Maintaining a healthy body weight in the long term, such as by adhering to a healthy lifestyle, may be beneficial to neurocognitive health.

## INTRODUCTION

Dementia is the leading causes of cognitive dysfunction and disability in older adults and affects an estimated 50 million people globally [1]. Several potentially modifiable factors may contribute to the prevention of dementia during the life course, including obesity, physical activity, and other lifestyle factors [1]. For example, having overweight or obesity in midlife, as measured by body mass index (BMI) and waist circumference (WC), are reported to be associated with increased risk of developing dementia and related risk factors such as hypertension, depression, and diabetes [2, 3, 4]. However, evidence from observational studies that have supported these findings is mainly based on single anthropometric measurements with the use of traditional cut points, without considering potential changes in obesity levels over time [3, 4]. Considering that most cases of dementia are characterized by pathophysiological changes over 20 years, as well as the dynamic nature of obesity biomarkers [5], it is critical to consider risk factors for dementia from a life‐span perspective [1]. Obesity in midlife may predict dementia, possibly attributable to vascular mechanisms, whereas weight usually falls after midlife in those with or who are developing dementia [6, 7], reflecting neurodegeneration and disruption of homeostatic feedback mechanisms in late life [8].

Several retrospective case–control studies that have examined longitudinal BMI trajectories before dementia diagnosis have suggested that older adults with dementia have a higher BMI in midlife and a significantly lower BMI in late life, starting from around 3 to 10 years prior to diagnosis [9, 10], with some studies having also reported a positive association or no association between obesity and dementia in late life [9, 11]. Although these findings highlight the importance of serial assessments of BMI beyond single measures, several key issues remain. First, the conflicting findings may be attributable to reverse causation due to weight loss during the preclinical and prodromal phase of dementia [7], thus leading to biased association. Prospective, population‐based cohorts with longitudinal anthropometric measures through middle to late life are needed to better clarify the temporal association. Second, evidence on the association between obesity trajectories and cognitive function is lacking. Mild cognitive impairment as a reversible state before dementia may provide an opportunity to prevent or delay dementia onset [12]. Third, previous studies have predominantly focused on BMI as a marker of general obesity, neglecting the characterization of body fat distribution. WC, a marker of the central form of obesity (e.g., visceral), may be a more sensitive marker than BMI, particularly in older individuals [13, 14]. However, the association of WC trajectories and subsequent dementia risk remains unclear. Finally, evidence has suggested that cumulative exposure to risk factors for dementia, such as blood pressure in midlife [15], is associated with subsequent cognitive impairments and development of dementia, whereas few studies have investigated the health effect of cumulative obesity. Overall, studies assessing the long‐term trajectory of both general and central obesity beyond midlife are critical to provide insight into the association among obesity, weight control, and subsequent cognitive function and dementia.

Based on a nationally representative, longitudinal, community‐based cohort, we aim to investigate the prospective association of longitudinal obesity trajectories throughout middle and late life with subsequent cognitive decline and dementia among cognitively healthy adults aged 50 years and older. These findings could have significant implications for developing targeted strategies aimed at reducing dementia risk that are tailored to an individual's long‐term obesity level. We hypothesize that consistently high patterns of both general and central obesity during midlife may be linked to the risk of developing dementia and experiencing cognitive decline later in life.

## METHODS

### Study population

The present study used data from the English Longitudinal Study of Ageing (ELSA), a longitudinal cohort comprising a representative sample of adults aged 50 years and older residing in private households in England, as has been described elsewhere [16]. The original sample comprised 11,391 participants from the Health Survey for England (HSE) conducted in 1998, 1999, and 2001 (wave 0), with additional refreshment samples drawn from the HSE at various waves (waves 3, 4, 6, 7, and 9) to adjust for the aging profile of the original sample. Data collection commenced on March 1, 2002, with subsequent assessments conducted every 2 years (from wave 2 to wave 9), capturing changes in health, economic status, and social circumstances through face‐to‐face interviews, self‐administered questionnaires, and nurse visits conducted every 4 years. This longitudinal study used data from waves 0, 2, 4, 6, and 9 of the ELSA. Trained nurses measured anthropometric indices at waves 0, 2, 4, and 6, which were then used to model obesity trajectories. Wave 6 (2012–2013) served as the baseline, with assessments of cognitive decline and dementia conducted from wave 7 (2014–2015) to wave 9 (2018–2019). Participants provided written informed consent, and ethical approval was obtained from the National Research Ethics Service. Analyses included individuals with anthropometric measures available at two or more of the four waves used for trajectory construction. Participants diagnosed with dementia or other memory impairments at baseline, as well as those lacking cognitive function assessments during follow‐up, were excluded. The study adhered to the Strengthening the Reporting of Observational Studies in Epidemiology (STROBE) reporting guidelines. Detailed study design and timelines are presented in Figure S1.

### Anthropometric measurements

At four separate time points (wave 0, 2, 4, and 6), trained nurses conducted measurements of height, weight, and WC. Height measurements were taken without shoes using a portable stadiometer placed horizontally in accordance with the Frankfort plane. Weight was measured using a portable electronic scale. BMI was calculated by dividing weight in kilograms by height in meters squared. WC was measured twice, with the midpoint between the lower rib and the upper margin of the iliac crest as the reference point. If the difference between the initial two WC measurements was within 3 cm, the mean value was used. However, if the difference exceeded 3 cm, a third measurement was taken, and the mean of the two closest results was calculated for analysis.

### Cognitive function and dementia

Cognitive function was evaluated through memory, orientation, and executive function tests, all of which have demonstrated validity and consistency. Memory was assessed using immediate and delayed word recall tests [17] during which participants were presented with 10 unrelated words and were asked to recall them immediately and after a short delay. Orientation was measured using a date‐naming test, which involved questions regarding the time, day, month, and year [15]. Executive function was assessed using an animal‐naming fluency test [18] during which participants were instructed to name as many animals as possible within a minute. Higher scores in these tests indicated better cognitive performance. In order to determine the rate of cognitive decline, scores from each cognitive domain were standardized to the cohort and then averaged to generate a standardized global cognitive score. This approach aligns with methodologies used in previous studies within the same ELSA cohort [15].

Dementia was defined using a triangulation approach involving three sources of information. First, self‐reported physician diagnoses of dementia or Alzheimer's disease by participants directly involved in the study were considered. Second, dementia was identified based on both cognitive and functional impairment [15, 19]. Cognitive impairment was defined as a score 1.5 standard deviations (SD) below the mean of the population, stratified by education level. Functional impairment was characterized by difficulty in independently performing one or more activities of daily living. Third, an adapted short‐form Informant Questionnaire on Cognitive Decline in the Elderly (IQCODE) questionnaire was completed by informants (family members or long‐term caregivers). This questionnaire compared the individual's present cognitive and functional performance with their performance from the previous 2 years [20]. A threshold of ≥3.38 was used to define dementia, with high sensitivity (0.82) and specificity (0.84) [20]. These methods for defining dementia have been validated within the same ELSA cohort [14, 15, 19], adhering to criteria from the *Diagnostic and Statistical Manual of Mental Disorders* (Fourth Edition) (DSM‐IV).

### Covariates

The models were adjusted for various covariates measured at baseline (wave 6), including age, sex, ethnicity, marital status, employment status, educational attainment, comorbidities (self‐reported physician‐diagnosed chronic lung disease, asthma, arthritis, osteoporosis, cancer, hypertension, and diabetes), lifestyle factors (smoking status, alcohol consumption, physical activity, and sleep duration), and mental health conditions (history of psychiatric disorders and depressive symptoms). Education levels were categorized into the following three tiers: low (below secondary); middle; and high (university or above). Depressive symptoms were assessed using an abbreviated eight‐item version of the validated Center for Epidemiologic Studies Depression Scale (CES‐D) [21]. Additionally, polygenic risk scores that captured an individual's load of common genetic variants associated with Alzheimer's disease were constructed and adjusted for in the sensitivity analysis. Participants with missing data on study exposures at baseline were excluded (percentage of all missing covariables was below 5%).

In order to further explore the impact of lifestyle factors among participants with obesity trajectories at high risk of developing dementia, individuals were categorized based on a composite healthy lifestyle score. This score integrated four modifiable healthy lifestyle factors and categories were determined in accordance with previous evidence and UK National Health Service (NHS) guidelines [22]. One point was assigned for each unhealthy lifestyle category, which included being a current smoker, consuming alcohol more than four times per week, engaging in moderate or vigorous physical activity less than once per week, and having a sleep duration of less than 7 or more than 9 h/day. Participants' scores were summed to create an unweighted score, which was then classified into favorable (score 0–1) and unfavorable (score 2–4) lifestyle categories.

### Statistical analysis

Latent mixture models were employed to discern subgroups sharing similar underlying trajectories of both general and central obesity, as measured by BMI and WC, respectively [23]. A censored normal model, suitable for continuous outcomes, was used to estimate multiple trajectories [24]. Through maximum likelihood estimation, various models incorporating different numbers and forms of potential patterns (such as intercept, linear, or quadratic slope) were fitted and assessed, with model fit assessed by the Bayesian information criterion (BIC). This approach facilitated handling missing data for each individual and yielded asymptotically unbiased parameter estimates. The determination regarding the number and shape of trajectories was based on a combination of prior knowledge (interpretation of trajectories) and statistical inference (model fit). Models were initiated with five classes, employing a quadratic form for all trajectory classes, followed by comparisons of BIC with models having four, three, two, and one classes. The model featuring five classes for BMI and four classes for WC exhibited superior fit, after which BIC was compared among models with different functional forms. Finally, the BMI model comprised five classes with up to quadratic order terms, whereas the WC model included linear order terms. Discrimination testing was conducted by computing the mean posterior estimated probability of final group membership.

In order to evaluate the association between obesity trajectories and subsequent cognitive decline, linear mixed models were employed. Years since baseline (wave 6) served as the time scale, with random intercept and slope at the individual level. An unstructured correlation matrix and a multivariate normal joint distribution were used. The models included obesity trajectory groups; time in years since baseline; and various covariates, including age, sex, ethnicity, marital status, employment, education, comorbidities, lifestyle factors, and mental health conditions. Continuous covariates were centered at the cohort median (age 57 years) or mean (depressive symptoms CES‐D score 0.72). Because the linear mixed model could appropriately handle observations of the dependent variable that were missing at random (such as cognition scores), no additional imputation procedures were implemented.

In order to evaluate the association between obesity trajectory groups and the risk of developing incident dementia, Cox regression models were used to estimate hazard ratios (HR) and 95% confidence intervals (CI). The time to event was measured from the baseline survey to the time when an individual was initially diagnosed with dementia or censored. Adjusted models incorporated a series of covariates similar to those used in the aforementioned linear mixed models. Proportional hazards assumptions were evaluated using tests of Schoenfeld residuals and log–log inspection, with no violations noted. Subgroup analyses were conducted to explore potential statistical interactions based on age (<65 vs. ≥65 years), sex, hypertension status (yes vs. no), and physical activity level (moderate/vigorous activity ≥1 time per week or <1 time per week).

In order to investigate the potential contribution of lifestyle and genetic factors to trajectory profiles, odds ratios (OR) for each trajectory relative to the lowest trajectory by genetic (high vs. low risk) and lifestyle (unfavorable vs. favorable) categories were calculated using logistic regression models, with adjustment for covariates [23].

Multiple sensitivity analyses were conducted to assess the robustness of the main analyses. First, in order to account for potential reverse causality wherein preclinical dementia may influence body weight, analyses were performed after excluding participants who developed dementia within the first 2 years of follow‐up. Second, additional adjustments were made for baseline WC in the association between BMI trajectories and dementia and vice versa for the association with WC trajectories. BMI and WC measurements complement each other in capturing different aspects of body composition, thereby providing a more comprehensive assessment of obesity. Third, in order to determine whether the association between obesity trajectories and dementia could be attributed solely to a single measure of BMI or WC, adjustments were made for BMI at wave 6 (i.e., start of follow‐up) in the association with dementia and similarly for WC. Finally, additional adjustments were made for genetic risk factors associated with dementia in the Cox regression model.

All analyses were conducted using SAS version 9.4 (SAS Institute Inc.) and R version 4.2.2 (R Foundation for Statistical Computing). All statistical tests were two‐sided, with a significance level set at *p* < 0.05.

## RESULTS

### Population characteristics

Of the 11,205 participants with available data collected at wave 0, 2, 4, and 6 of the ELSA, 73.2% had at least two anthropometric measures out of the four waves. A total of 4751 eligible participants who were cognitively healthy at wave 6 baseline and had at least one assessment of cognitive function and dementia during subsequent follow‐up waves were included in the study (Figure S1). Of these, the mean (SD) age was 69.2 (14.0) years, with 2044 (43.0%) being male and 4521 (95.2%) identifying as White. The median follow‐up duration for the study outcomes was 6 years (interquartile range, 4–6 years).

### General and central obesity trajectory patterns

**FIGURE 1:**
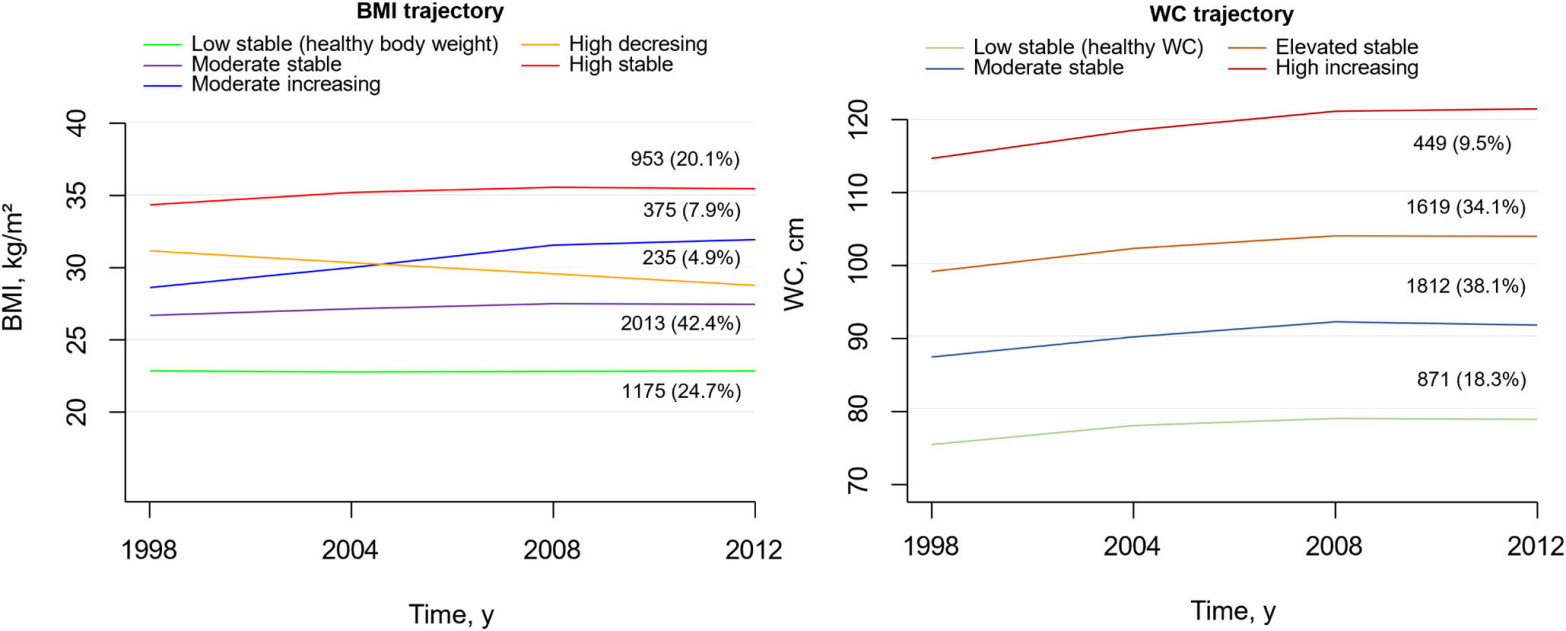
Trajectories in general and central obesity as measured by BMI and waist circumference (WC) from 1998 to 2012. Trajectory groups of general and central obesity measured by BMI and WC, patterns by time, and number of participants in each class. During the 14‐year span, trajectories for BMI revealed the following distinct patterns: a low‐stable BMI pattern, ranging from 22.8 to 22.9 kg/m^2^ and indicating stable healthy body weight; a moderate‐stable pattern ranging from 26.6 to 27.4 kg/m^2^ and representing stable overweight; a moderate‐increasing pattern displaying a mean increase from 28.4 to 32.5 kg/m^2^ and indicating transition from overweight to obesity; a high‐decreasing pattern displaying a mean decrease from 31.5 to 28.6 kg/m^2^ and indicating transition from obesity to overweight; and a high‐stable pattern ranging from 34.3 to 35.4 kg/m^2^ and representing stable obesity. WC trajectories included a low‐stable WC pattern ranging from 76 to 79 cm and representing healthy WC, a moderate‐stable pattern ranging from 88 to 92 cm, an elevated‐stable pattern ranging from 100 to 105 cm, and a high‐increasing pattern ranging from 115 to 122 cm.

Over a 14‐year period spanning middle age, distinct trajectories for both BMI and WC were delineated, as depicted in Figure 1. For general obesity measured by BMI, five discrete trajectories were identified: a low‐stable pattern ranging from 22.9 to 22.8 kg/m^2^ (*n* [percentage], 1175 [24.7%], indicative of stable healthy body weight); a moderate‐stable pattern ranging from 26.6 to 27.4 kg/m^2^ (*n* [percentage], 2013 [42.4%], reflecting stable overweight); a moderate‐increasing pattern, characterized by a mean increase from 28.4 to 32.5 kg/m^2^ (*n* [percentage], 375 [7.9%], transitioning from overweight to obesity); a high‐decreasing pattern, with a mean decrease from 31.5 to 28.6 kg/m^2^ (*n* [percentage], 238 [4.9%], shifting from obesity to overweight); and a high‐stable pattern ranging from 34.3 to 35.4 kg/m^2^ (*n* [percentage], 953 [20.1%], representing stable obesity). The mean (SD) probabilities for each individual's final group membership ranged from 0.95 (0.07) to 0.98 (0.06) across the BMI trajectory groups.

For central obesity as measured by WC, the following four distinct trajectories were identified: a low‐stable pattern ranging from 76 to 79 cm (*n* [percentage], 871 [18.3%], indicative of a healthy WC); a moderate‐stable pattern ranging from 88 to 92 cm (*n* [percentage], 1812 [38.1%]); an elevated‐stable pattern ranging from 100 to 105 cm (*n* [percentage], 1619 [34.1%]); and a high‐increasing pattern ranging from 115 to 122 cm (*n* [percentage], 449 [9.5%]). The mean (SD) probabilities for each individual's final group membership ranged from 0.91 (0.13) to 0.94 (0.12) across the WC trajectory groups. The baseline characteristics of participants categorized by BMI and WC trajectories are detailed in Table 1 and Table S1, respectively.

**TABLE 1:** Baseline characteristics by BMI trajectory group.

	Total (*n* = 4751)	BMI trajectory^a^					*p* value
		Low‐stable (*n* = 1175)	Moderate‐stable (*n* = 2013)	Moderate‐increasing (*n* = 375)	High‐decreasing (*n* = 235)	High‐stable (*n* = 953)	
Age, mean (SD), y							
Wave 0 (start of trajectory)	58.7 (8.1)	58.6 (8.5)	59.0 (7.9)	57.3 (8.0)	61.8 (8.8)	58.0 (7.6)	<0.001
Wave 6 (baseline)	69.2 (14.0)	67.9 (16.2)	69.8 (13.4)	68.6 (12.3)	69.9 (18.9)	69.5 (11.3)	<0.01
Sex, *n* (%)							<0.001
Male	2044 (43.0)	424 (36.1)	1017 (50.5)	146 (38.9)	96 (40.9)	361 (37.9)	
Female	2707 (57.0)	751 (63.9)	996 (49.5)	229 (61.1)	139 (59.1)	592 (62.1)	
Ethnicity, *n* (%)							0.81
White	4521 (95.2)	1123 (95.6)	1918 (95.3)	355 (94.7)	227 (96.6)	898 (94.2)	
Non‐White	107 (2.3)	22 (1.9)	45 (2.2)	9 (2.4)	5 (2.1)	26 (2.7)	
Employment, *n* (%)							0.01
Retired	3618 (76.2)	895 (76.2)	1551 (77.0)	264 (70.4)	192 (81.7)	716 (75.1)	
Employed	682 (14.4)	172 (14.6)	295 (14.7)	70 (18.7)	23 (9.8)	122 (12.8)	
Unemployed, sick, disabled, and other	328 (6.9)	78 (6.6)	117 (5.8)	30 (8.0)	17 (7.2)	86 (9.0)	
Marriage, *n* (%)							<0.001
Single	204 (4.3)	76 (6.5)	70 (3.5)	11 (2.9)	7 (3.0)	40 (4.2)	
Married or remarried	2916 (61.4)	696 (59.2)	1298 (64.5)	229 (61.1)	132 (56.2)	561 (58.9)	
Separated	32 (0.8)	11 (0.9)	16 (0.8)	4 (1.1)	1 (0.4)	7 (0.7)	
Divorced	481 (10.1)	135 (11.5)	189 (9.4)	43 (11.5)	20 (8.5)	94 (9.9)	
Widowed	977 (20.6)	225 (19.1)	387 (19.2)	77 (20.5)	69 (29.4)	219 (23.0)	
Educational attainment, *n* (%)							<0.001
Low (below secondary)	2273 (47.8)	490 (41.7)	917 (45.6)	204 (54.4)	136 (57.9)	526 (55.2)	
Middle	1588 (33.4)	404 (34.4)	714 (35.5)	122 (32.5)	68 (28.9)	280 (29.4)	
High (university or above)	750 (15.8)	244 (20.8)	326 (16.2)	37 (9.9)	28 (11.9)	115 (12.1)	
BMI, mean (SD), kg/m^2^							
Year 1998	27.6 (4.5)	22.9 (1.9)	26.6 (1.8)	28.4 (1.7)	31.5 (1.5)	34.3 (3.7)	<0.001
Year 2004	28.0 (4.9)	22.8 (1.9)	27.1 (1.9)	30.1 (1.9)	30.5 (2.0)	35.1 (4.1)	<0.001
Year 2008	28.3 (5.1)	22.8 (1.9)	27.4 (2.1)	32.0 (1.9)	29.6 (2.2)	35.5 (4.3)	<0.001
Year 2012	28.3 (5.1)	22.8 (2.1)	27.4 (1.9)	32.5 (2.1)	28.6 (2.3)	35.4 (4.3)	<0.001
WC, mean (SD), cm							
Year 1998	92.2 (12.9)	80.6 (8.7)	91.2 (9.3)	94.1 (8.9)	101.0 (9.2)	105.7 (11.4)	<0.001
Year 2004	95.2 (13.0)	82.6 (8.3)	94.3 (8.7)	100.5 (9.2)	101.3 (9.3)	109.8 (10.7)	<0.001
Year 2008	97.1 (13.4)	83.3 (8.4)	96.3 (8.5)	105.5 (9.3)	100.9 (9.4)	111.6 (11.2)	<0.001
Year 2012	96.6 (13.6)	83.2 (8.8)	95.7 (9.0)	106.4 (9.6)	98.7 (9.5)	111.2 (11.6)	<0.001
Sleep duration, mean (SD), h	6.9 (1.3)	6.9 (1.3)	6.9 (1.3)	6.7 (1.4)	6.9 (1.5)	6.8 (1.4)	0.01
Healthy lifestyle factors, *n* (%)							
No current smoking (past or never smoker)	4184 (88.1)	977 (83.1)	1813 (90.1)	332 (88.5)	211 (89.8)	851 (89.3)	<0.001
Moderate alcohol consumption (≤4 times/wk)	2992 (63.0)	734 (62.5)	1250 (62.1)	231 (61.6)	164 (69.8)	613 (64.3)	0.36
Regular physical activity (moderate or vigorous activity ≥ 1 time/wk)	3404 (71.6)	897 (76.3)	1522 (75.6)	253 (67.5)	150 (63.8)	582 (61.1)	<0.001
Adequate sleep duration (7–9 h/d)	2666 (56.1)	695 (59.1)	1151 (57.2)	198 (52.8)	134 (57.0)	488 (51.2)	0.02
Comorbidity, *n* (%)							
Chronic lung disease	199 (4.2)	60 (5.1)	60 (3.0)	23 (6.1)	9 (3.8)	47 (4.9)	0.03
Asthma	483 (10.2)	103 (8.8)	186 (9.2)	47 (12.5)	25 (10.6)	122 (12.8)	0.03
Arthritis	1901 (40.0)	384 (32.7)	741 (36.8)	153 (40.8)	111 (47.2)	512 (53.7)	<0.001
Osteoporosis	372 (7.8)	128 (10.9)	138 (6.9)	27 (7.2)	21 (8.9)	58 (6.1)	<0.01
Cancer	164 (3.5)	42 (3.6)	62 (3.1)	14 (3.7)	4 (1.7)	42 (4.4)	0.37
Hypertension	1855 (39.0)	308 (26.2)	761 (37.8)	174 (46.4)	106 (45.1)	506 (53.1)	<0.001
Diabetes	497 (10.5)	52 (4.4)	165 (8.2)	42 (11.2)	32 (13.6)	206 (21.6)	<0.001
Baseline mental health conditions, *n* (%)							
History of diagnosed mental disorders	391 (8.2)	88 (7.5)	159 (7.9)	43 (11.5)	12 (5.1)	89 (9.3)	0.08
Symptoms of depression (CES‐D ≥ 4)	529 (11.1)	125 (10.6)	188 (9.3)	49 (13.1)	36 (15.3)	131 (13.7)	0.01
Posterior estimated probability of membership, mean (SD)	NA	0.97 (0.09)	0.96 (0.11)	0.97 (0.06)	0.95 (0.07)	0.98 (0.06)	‐

*Note*: Data are mean (SD) or *n* (%) for continuous and categorical variables, as appropriate. Data are missing for ethnicity for 123 participants (2.6%), for marriage for 134 participants (2.8%), for education for 140 participants (2.9%), for smoking for 123 participants (2.6%), for sleep duration for 235 participants (4.9%), and for comorbidities for 123 participants (2.6%).

Abbreviations: CES‐D, Center for Epidemiologic Studies Depression Scale; NA, not applicable; WC, waist circumference.

^a^ The low‐stable BMI pattern ranged from 22.8 to 22.9 kg/m^2^ (stable healthy body weight); the moderate‐stable pattern ranged from 26.6 to 27.4 kg/m^2^ (stable overweight); the moderate‐increasing pattern had a mean increase from 28.4 to 32.5 kg/m^2^ (overweight to obesity); the high‐decreasing pattern had a mean decrease from 31.5 to 28.6 kg/m^2^ (obesity to overweight); and the high‐stable pattern had a range from 34.3 to 35.4 kg/m^2^ (stable obesity).

The relationship of genetic and lifestyle factors and each obesity trajectory compared with the low‐stable (healthy) pattern are shown in Table S2. Relative to the low‐stable trajectory, both general and central obesity trajectories exhibited significant associations with genetic factors in a dose–response manner, particularly evident in the high‐stable/increasing groups (e.g., high‐stable BMI pattern, OR, 4.31, 95% CI: 3.28–5.73; high‐increasing WC pattern, OR, 3.91, 95% CI: 2.75–5.58). Additionally, high/moderate‐increasing obesity trajectories were also related to lifestyle factors (e.g., for unfavorable vs. favorable lifestyle category, high‐stable BMI group, OR, 1.30, 95% CI: 1.07–1.57; high‐increasing WC group, OR, 1.80, 95% CI: 1.37–2.34).

### General and central obesity trajectory and cognitive decline

**FIGURE 2:**
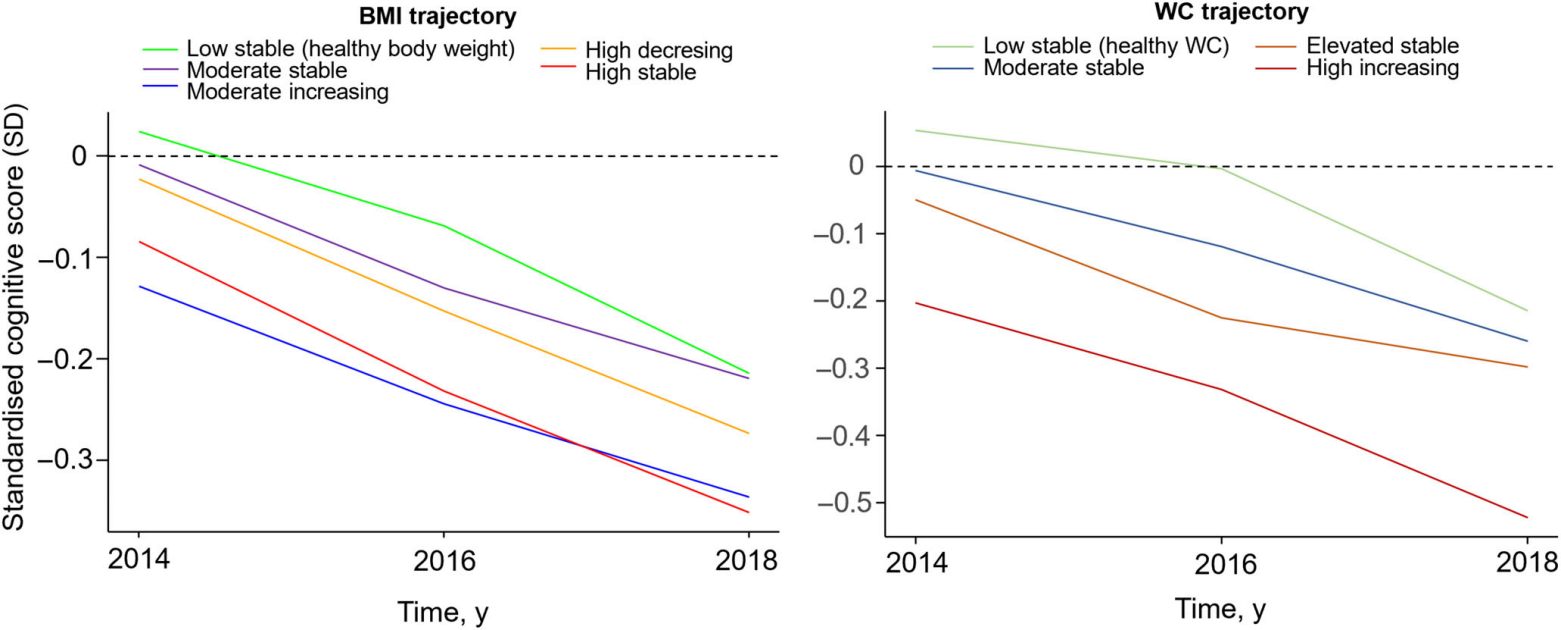
Cognitive function by trajectory groups of BMI and waist circumference (WC) through midlife to late life. Results were based on a linear mixed model adjusted for sociodemographic and socioeconomic characteristics, comorbidities, lifestyle factors, and depressive symptoms. Data are plotted based on the reference values of covariates (male, married or remarried, education of university or above, no chronic conditions, no current smoking, moderate alcohol consumption [≤4 times/week], moderate or vigorous activity ≥ 1 time/week, and sleep duration of 7–9 h/day) or on the mean value (age and depressive symptoms [Center for Epidemiologic Studies Depression Scale (CES‐D) score]) in the cohort.

Rate of cognitive decline differed between groups of BMI and WC trajectories (Table 2; Figure 2). Relative to individuals maintaining a consistently healthy BMI in the low‐stable group, participants exhibiting moderate‐increasing and high‐stable BMI patterns experienced more pronounced declines in cognitive function (difference in 6‐year decline: −0.14 SD [95% CI: −0.25 to −0.04] for the moderate‐increasing group; −0.11 SD [95% CI: −0.18 to −0.03] for the high‐stable group). Similarly, compared with those with consistently low WC measurements, individuals with moderate‐stable and high‐increasing WC patterns demonstrated inferior cognitive performance (−0.09 SD [95% CI: −0.16 to −0.02] for the moderate‐stable group; −0.18 SD [95% CI: −0.28 to −0.07] for the high‐increasing group).

**TABLE 2:** Association of general and central trajectory over 14 years beyond midlife with cognitive decline and incident dementia.

	Cognitive decline		Dementia				
	β (95% CI), *p* value^a^		Events (%)	HR (95% CI), *p* value^b^			
				Age‐ and sex‐adjusted		Multivariable‐adjusted	
General obesity, BMI trajectory^c^							
Low‐stable	Reference		83 (7.1)	1 (Reference)		1 (Reference)	
Moderate‐stable	−0.03 (−0.10 to 0.03)	0.29	170 (8.5)	1.26 (0.97 to 1.65)	0.08	1.32 (0.98 to 1.76)	0.09
Moderate‐increasing	−0.14 (−0.25 to −0.04)	0.01	28 (7.5)	1.30 (0.84 to 1.99)	0.23	1.19 (0.74 to 1.93)	0.53
High‐decreasing	−0.02 (−0.16 to 0.09)	0.58	27 (11.5)	1.20 (0.78 to 1.86)	0.40	1.22 (0.76 to 1.96)	0.59
High‐stable	−0.11 (−0.18 to −0.03)	<0.01	87 (9.1)	1.57 (1.16 to 2.13)	<0.01	1.43 (1.02 to 2.00)	0.04
Central obesity, WC trajectory^c^							
Low‐stable	Reference		53 (6.1)	1 (Reference)		1 (Reference)	
Moderate‐stable	−0.09 (−0.16 to −0.02)	0.02	164 (9.1)	1.50 (1.10 to 2.05)	0.01	1.45 (1.06 to 1.99)	0.01
Elevated‐stable	−0.06 (−0.14 to 0.01)	0.11	143 (8.8)	1.54 (1.11 to 2.15)	0.01	1.51 (1.08 to 2.12)	0.02
High‐increasing	−0.18 (−0.28 to −0.07)	<0.001	35 (7.8)	1.91 (1.22 to 2.98)	<0.01	1.57 (1.01 to 2.49)	0.03

Abbreviations: HR, hazard ratio; WC, waist circumference.

^a^ Results are based on linear mixed model adjusted for age, sex, ethnicity, marital status, employment, education, comorbidities, lifestyle factors, and mental health conditions.

^b^ Models were adjusted for age, sex, ethnicity, marital status, employment, education, comorbidities, lifestyle factors, and mental health conditions.

^c^ From 1998 to 2012, five distinct BMI trajectories were identified, including the low‐stable BMI pattern, which ranged from 22.8 to 22.9 kg/m^2^ (stable healthy body weight); the moderate‐stable pattern, which ranged from 26.6 to 27.4 kg/m^2^ (stable overweight); the moderate‐increasing pattern, with a mean increase from 28.4 to 32.5 kg/m^2^ (overweight to obesity); the high‐decreasing pattern, with a mean decrease from 31.5 to 28.6 kg/m^2^ (obesity to overweight); and the high‐stable pattern, which ranged from 34.3 to 35.4 kg/m^2^ (stable obesity). Four distinct WC trajectories were identified, including the low‐stable WC pattern, which ranged from 76 to 79 cm (healthy WC); the moderate‐stable pattern, which ranged from 88 to 92 cm; the elevated‐stable pattern, which ranged from 100 to 105 cm; and the high‐increasing pattern, which ranged from 115 to 122 cm.

### General and central obesity trajectory and dementia

The association between obesity trajectories and subsequent risk of developing dementia are present in Table 2. Over a median follow‐up of 6 years, 395 incident dementia cases were identified. Participants in the high‐stable BMI group, compared with those in the low‐stable BMI group who maintained a healthy body weight for 14 years beyond middle age, were at a significantly increased risk of developing dementia (HR, 1.43, 95% CI: 1.02–2.00), following adjustment for potential confounders. Similarly, compared with individuals in the low‐stable WC group, those in the moderate‐stable (HR, 1.45, 95% CI: 1.06–1.99), elevated‐stable (HR, 1.51, 95% CI: 1.08–2.12), and high‐increasing (HR, 1.57, 95% CI: 1.01–2.49) trajectory patterns exhibited elevated risks of dementia. No significant interaction was observed for age, sex, hypertension, and physical activity (Tables S3 and S4). Sensitivity analyses, which excluded incident dementia cases occurring within the first 2 years of follow‐up and were additionally adjusted for genetic risk for dementia or excluded participants with cancer at baseline, yielded consistent results regarding the association between BMI and WC trajectories and the risk of developing dementia (Tables S5 and S6). Further adjustments for a single measure of WC or BMI at baseline did not materially alter the results for BMI trajectories. However, the association between WC trajectories and dementia was attenuated following further adjustment for a single BMI or WC measured at baseline.

## DISCUSSION

Based on a nationally representative, community‐based cohort with repeated measures, we found that longitudinal trajectories of both general and central obesity beyond middle age were significantly associated with subsequent cognitive decline and the risk of developing incident dementia in late life, even after adjustment for single obesity measures at baseline. Over a 14‐year span from middle to late life, we identified five distinct BMI trajectories and four WC trajectories.

These distinct trajectories were driven by genetic factors in a dose–response manner, particularly evident in the high‐stable/increasing patterns, whereas high/moderate‐increasing patterns were also influenced by lifestyle factors. Participants in the high‐stable BMI trajectory, indicative of sustained obesity, experienced a more rapid rate of cognitive decline and increased risk of dementia in late life compared with those in the low‐stable BMI trajectory, representing a consistently healthy body weight. The moderate‐stable BMI trajectory was associated with accelerated cognitive decline, but not incident dementia. Moreover, WC trajectory patterns consistently correlated with subsequent cognitive decline and dementia risk, with the highest risk observed in the high‐increasing WC group. The moderate‐stable WC trajectory was linked to both rapid cognitive decline and incident dementia. Central obesity trajectories beyond middle age, reflecting long‐term central adiposity, emerged as a more sensitive marker associated with cognitive function and dementia compared with BMI trajectories reflecting general obesity. These findings underscore the importance of managing weight beyond middle age, including adhering to a healthy lifestyle, to promote neurocognitive health and overall well‐being.

Previous studies that have been based on single anthropometric measures have yielded inconsistent results [9, 10, 11, 25, 26, 27, 28, 29, 30, 31]. Although most studies have reported that having obesity or overweight in midlife was associated with increased risk of developing dementia [26], the current largest cohort of 2 million participants reported that individuals with overweight in middle age (median, 40–55 years) were at lower risk of developing dementia throughout two decades of follow‐up [25]. Current evidence more consistently suggested that having overweight in late life was related to decreased dementia risk [25, 26]. The controversial findings were partly attributable to reverse causality in which weight loss occurred during the long incubation period of dementia, as noted in several [9, 10], but not all [9, 11], case–control studies, and significantly lower BMI occurs ~3 to 10 years before diagnosis of dementia or mild cognitive impairment. These findings suggest that the association between obesity and dementia is likely to depend on when obesity biomarkers are measured in the life course [29], highlighting the importance of longitudinal anthropometric assessment throughout middle to late life, beyond single measures at a fixed time point, to further clarify the association of interest. However, previous studies have typically measured BMI at one time point (although several studies have stratified by baseline age group); limited studies have examined the effect of BMI change at two time points [27, 28]; and none, to our knowledge, has considered the longitudinal patterns of BMI, as well as WC, during a prolonged period throughout middle and late life.

The findings regarding the association of the high‐stable BMI trajectory, representing a prolonged pattern of obesity (range, 34.3–35.4 kg/m^2^ over 14 years), with dementia are partially supported by an individual‐level meta‐analysis of 1.3 million adults showing that BMI is associated with increased risk of developing dementia when BMI was measured >20 years before diagnosis, and the follow‐up was long (HR, 1.16, 95% CI: 1.05–1.27 per 5‐kg/m^2^ increase in BMI) [32]. The present study, with a median time of 20 years between the start of the trajectory and dementia diagnosis, extends those finding to demonstrate that not only is a single BMI in middle age important, but certain obesity trajectories were associated with the risk of developing dementia. In addition, the observed associations could not be totally explained by a single measure at the start of the trajectory, suggesting that the persistent “exposure” to high BMI level beyond middle age, similar to other risk factors for dementia such as systolic blood pressure [15], may have cumulative long‐term effects. By contrast, we did not identify a significant association with high‐decreasing BMI patterns, although previous studies have suggested that a single BMI measure close to diagnosis was inversely related to dementia risk, likely driven by reverse causality [3]. A recent study also reported that the pattern of increased BMI in early midlife followed by decline in late life, but not consistent BMI decline from middle to late life, was associated with increased risk of developing dementia [33]. Nevertheless, the limited sample size of the high‐decreasing group warranted further investigation with a larger sample and longer follow‐up. Coupled with increased risk of developing dementia, we observed that BMI trajectories such as moderate‐increasing and high‐stable patterns were associated with more rapid cognitive decline, an early sign of dementia. Previous studies have similarly suggested that a single BMI measure in midlife as well as significant BMI change at two points (defined as increase or decrease of ≥5%, indicating higher variability) was related to faster cognitive decline [26, 27]. These findings collectively indicated the importance of weight management beyond middle age to promote neurocognitive health and help prevent the progression of cognitive impairment and dementia.

Compared with the association with general obesity defined by BMI trajectories, we found that central obesity, as measured by longitudinal patterns of stable higher WC beyond middle age, was more consistently associated with accelerated cognitive decline and the risk of developing dementia in late life, which is supported by previous studies of single midlife WC measures [34]. As a simple indicator of weight relative to height, BMI does not distinguish between fat and lean body mass, which tend to change with aging. For example, participants with normal BMI but higher WC were at a significantly higher risk of developing dementia compared with those without central obesity [13]. These results suggest that WC provides additional information regarding the risk of developing cognitive decline and dementia that cannot be captured by BMI, although the two obesity biomarkers are intrinsically correlated.

Cumulatively, long‐term distinct trajectories in both general and central obesity may be useful to distinguish individuals at risk of developing cognitive impairment and dementia more accurately than single anthropometric measures. For example, although high‐stable and high‐decreasing BMI groups had a similar starting level of obesity during middle age, we found that the stable obesity pattern, but not the decreasing pattern from obesity to overweight, was associated with cognitive decline and dementia. If the identified associations with obesity trajectories are causal, targeted interventions across the life course are needed to protect against dementia in individuals with high‐risk obesity profiles. Overall, our findings suggest that long‐term maintenance of healthy weight and body composition beyond middle age, e.g., by adherence to a healthy lifestyle such as by engaging in physical activity and getting adequate sleep, may be beneficial for cognitive function. These are consistent with current guidelines for dementia prevention, which include maintaining cognitive, physical, and social activity in both midlife and late life [1].

By leveraging a nationally representative, community‐based cohort with standardized data collection over two decades spanning middle and late life, we explored longitudinal patterns of obesity and prospectively examined their association with subsequent cognitive decline and dementia in late life. The prospective design is less susceptible to reverse causality than previous retrospective case–control studies of BMI trajectories before dementia diagnosis. Our findings reveal robust associations between both general and central obesity trajectories over a 14‐year span, driven by genetic and lifestyle factors, and subsequent cognitive decline and dementia. However, several limitations should be acknowledged. First, the participants in ELSA were predominantly White adults from the UK; therefore, the identified trajectories and study findings may not be universally applicable to other populations despite the broad stratification of trajectories by conventional cut points of anthropometric measurements. Second, although we employed a triangulation approach to identify dementia cases, the possibility of undiagnosed disease cannot be entirely ruled out, potentially introducing misclassification bias. Nonetheless, such bias tends to bias risk estimates toward null, resulting in more conservative results if undiagnosed cases exhibit lower BMI values compared with diagnosed dementia cases. Third, despite adjusting for numerous potential confounders, employing a prospective design, and excluding events occurring within the first 2 years of follow‐up in sensitivity analyses, residual confounding and reverse causality remain possible due to the observational nature of the current study. Alternative study designs such as Mendelian randomization may be needed to clarify causality, although evidence from Mendelian randomization studies is currently inconsistent, and survival bias may further complicate the interpretation of results (e.g., individuals with a higher genetic risk of higher BMI may have an increased risk of death and thus be underrepresented among people with dementia) [35]. Fourth, the limited number of incident dementia cases restricted analyses by subcategories of dementia, such as Alzheimer's disease and vascular dementia. Fifth, we did not account for competing risk of death without information on mortality, which was likely to introduce potential bias. Sixth, we found no significant interaction in subgroup analyses, suggesting that the association of adiposity trajectories with outcomes is broadly consistent across population subgroups. However, several subgroups with low‐to‐modest sample size may have limited statistical power, which warrants further investigation with a larger sample size and additional covariables of interest. Finally, several trajectories such as the high‐decreasing and moderate‐increasing BMI trajectories were characterized by relatively small sample sizes, potentially limiting statistical power. Therefore, additional studies with larger sample sizes, longitudinal assessment of anthropometric measures, and long‐term follow‐up are necessary to validate and replicate the current findings.

## CONCLUSION

Distinct trajectories of general and central obesity throughout middle and late life driven by both genetic and lifestyle factors, particularly the consistently high patterns, are found to be linked with subsequent cognitive decline and the risk of developing dementia. These findings underscore the importance of longitudinally assessing obesity biomarkers beyond single anthropometric measures. Sustaining a healthy body weight over the long term and managing weight by adhering to a healthy lifestyle may contribute to better neurocognitive health and help prevent the progression of cognitive impairment and dementia. Further research is needed to elucidate causality and validate the predictive value of specific obesity trajectories. Additionally, exploring the impact of lifestyle modifications and interventions on adverse obesity trajectories and associated cognitive outcomes is warranted.

## AUTHOR CONTRIBUTIONS

Zhengting Liang and Yunhe Wang were responsible for the study design. Gang Hu and Yunhe Wang performed data collection, data analysis, and data interpretation. Gang Hu and Yunhe Wang drafted the manuscript. Gang Hu, Huibo Qin, Binbin Su, Yanping Bao, Zhengting Liang, and Yunhe Wang revised the manuscript and approved the final version.

## CONFLICT OF INTEREST STATEMENT

The authors declared no conflicts of interest.

## Supporting information


**Data S1.** Supporting information.

## Data Availability

The English Longitudinal Study of Ageing (ELSA) data were available through the UK Data Service (https://ukdataservice.ac.uk/). The authors thank the UK Data Service and ELSA for providing data.
