# Supplementary material for: Trajectories of general and central obesity beyond middle age in relation to late‐life cognitive decline and dementia
Source: Obesity (Silver Spring). 2025 Jan 28;33(2):405–15. doi: 10.1002/oby.24208 (PMC11774005; doi:10.1002/oby.24208)
Supplement: Supplementary file 1 — Data S1. Supporting information. [file OBY-33-405-s001.docx]

**Figure S1. Study design and timelines**
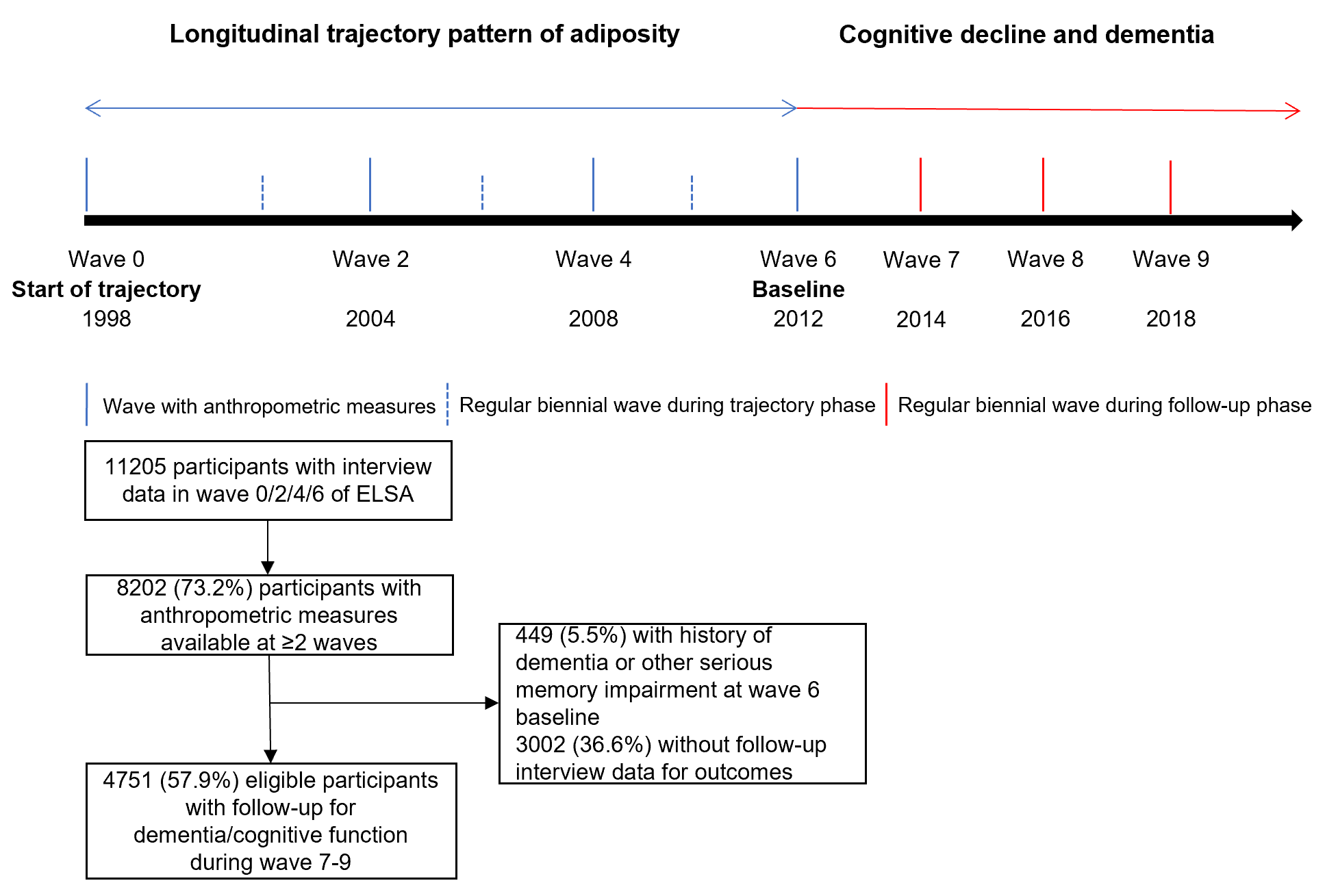


**Table S1. Baseline characteristics by WC trajectory group**

|  |  | **Total**  **(n=4751)** | **WC trajectory**^†^ | | | |  |
| --- | --- | --- | --- | --- | --- | --- | --- |
| **Characteristics**^#^ | | | **Low-stable**  **(n=871)** | **Moderate-stable (n=1812)** | **Elevated-stable (n=1619)** | **High-increasing**  **(n=449)** | **p value** |
| Age, mean (SD), y | |  |  |  |  |  |  |
| Wave 0 (start of trajectory) | | 58.7 (8.1) | 57.8 (8.1) | 58.9 (8.2) | 59.4 (8.1) | 57.1 (7.4) | <0.001 |
| Wave 6 (baseline) | | 69.2 (14.0) | 68.2 (14.2) | 68.9 (15.0) | 70.0 (13.7) | 69.1 (10.2) | 0.01 |
| Sex (%) | |  |  |  |  |  | <0.001 |
| Male | | 2044 (43.0) | 81 (9.3) | 647 (35.7) | 1021 (63.1) | 295 (65.7) |  |
| Female | | 2707 (57.0) | 790 (90.7) | 1165 (64.3) | 598 (36.9) | 154 (34.3) |  |
| Ethnicity (%) | |  |  |  |  |  | 0.12 |
| White | | 4521 (95.2) | 836 (96.0) | 1716 (94.7) | 1552 (95.9) | 417 (92.9) |  |
| Non-white | | 107 (2.3) | 13 (1.5) | 46 (2.5) | 33 (2.0) | 15 (3.3) |  |
| Employment (%) | |  |  |  |  |  | <0.01 |
| Retired | | 3618 (76.2) | 657 (75.4) | 1364 (75.3) | 1272 (78.6) | 325 (72.4) |  |
| Employed | | 682 (14.4) | 123 (14.1) | 259 (14.3) | 234 (14.5) | 66 (14.7) |  |
| Unemployed, sick, disabled, and others | | 328 (6.9) | 69 (7.9) | 139 (7.7) | 79 (4.9) | 41 (9.1) |  |
| Marriage (%) | |  |  |  |  |  | <0.01 |
| Single | | 204 (4.3) | 47 (5.4) | 74 (4.1) | 63 (3.9) | 20 (4.5) |  |
| Married or remarried | | 2916 (61.4) | 492 (56.5) | 1095 (60.4) | 1054 (65.1) | 275 (61.2) |  |
| Separated | | 39 (0.8) | 12 (1.4) | 11 (0.6) | 10 (0.6) | 6 (1.3) |  |
| Divorced | | 481 (10.1) | 108 (12.4) | 178 (9.8) | 153 (9.5) | 42 (9.4) |  |
| Widowed | | 977 (20.6) | 189 (21.7) | 396 (21.9) | 304 (18.8) | 88 (19.6) |  |
| Educational attainment (%) | |  |  |  |  |  | <0.001 |
| Low (below secondary) | | 2273 (47.8) | 354 (40.6) | 825 (45.5) | 845 (52.2) | 249 (55.5) |  |
| Middle | | 1588 (33.4) | 320 (36.7) | 637 (35.2) | 505 (31.2) | 126 (28.1) |  |
| High (university or above) | | 750 (15.8) | 170 (19.5) | 294 (16.2) | 231 (14.3) | 55 (12.2) |  |
| BMI, mean (SD), kg/m^2^ | |  |  |  |  |  |  |
| Year 2000 | | 27.5 (4.5) | 23.0 (2.3) | 26.3 (2.8) | 29.5 (3.3) | 35.1 (4.9) | <0.001 |
| Year 2004 | | 28.0 (4.9) | 23.0 (2.5) | 26.6 (2.9) | 30.0 (3.4) | 36.2 (5.2) | <0.001 |
| Year 2008 | | 28.3 (5.1) | 23.2 (2.6) | 26.9 (3.3) | 30.3 (3.4) | 37.1 (5.1) | <0.001 |
| Year 2012 | | 28.3 (5.1) | 23.1 (2.8) | 26.9 (3.2) | 30.4 (3.6) | 36.9 (5.3) | <0.001 |
| WC, mean (SD), cm | |  |  |  |  |  |  |
| Year 2000 | | 92.2 (12.9) | 75.5 (5.5) | 88.0 (5.9) | 100.2 (6.4) | 114.6 (8.6) | <0.001 |
| Year 2004 | | 95.2 (13.0) | 77.9 (5.5) | 90.7 (5.1) | 103.3 (5.6) | 118.5 (7.8) | <0.001 |
| Year 2008 | | 97.0 (13.4) | 79.1 (5.7) | 92.7 (5.3) | 105.1 (5.6) | 121.7 (8.1) | <0.001 |
| Year 2012 | | 96.6 (13.6) | 78.6 (6.1) | 92.1 (5.7) | 105.0 (5.8) | 121.9 (8.3) | <0.001 |
| Sleep duration, mean (SD), h | | 6.9 (1.3) | 6.9 (1.3) | 6.8 (1.3) | 6.9 (1.4) | 6.7 (1.4) | 0.04 |
| Healthy lifestyle factors, % | |  |  |  |  |  |  |
| No current smoking (past or never smoker) | | 4184 (88.1) | 733 (84.2) | 1599 (88.2) | 1461 (90.2) | 391 (87.1) | <0.001 |
| Moderate alcohol consumption (≤4 times/w) | | 2992 (63.0) | 535 (61.4) | 1159 (64.0) | 1013 (62.6) | 285 (63.5) | 0.32 |
| Regular physical activity (moderate or vigorous activity ≥1/w) | | 3404 (71.6) | 685 (78.6) | 1329 (73.3) | 1122 (69.3) | 268 (59.7) | <0.001 |
| Adequate sleep duration (7-9 h/d) | | 2666 (56.1) | 507 (58.2) | 1012 (55.8) | 928 (57.3) | 219 (48.8) | 0.02 |
| Comorbidity (%) | |  |  |  |  |  |  |
| Chronic lung disease | | 199 (4.2) | 34 (3.9) | 61 (3.4) | 74 (4.6) | 30 (6.7) | 0.02 |
| Asthma | | 483 (10.2) | 58 (6.7) | 187 (10.3) | 173 (10.7) | 65 (14.5) | <0.001 |
| Arthritis | | 1901 (40.0) | 304 (34.9) | 693 (38.2) | 680 (42.0) | 224 (49.9) | <0.001 |
| Osteoporosis | | 372 (7.8) | 121 (13.9) | 146 (8.1) | 85 (5.3) | 20 (4.5) | <0.001 |
| Cancer | | 164 (3.5) | 21 (2.4) | 63 (3.5) | 58 (3.6) | 22 (4.9) | 0.12 |
| Hypertension | | 1855 (39.0) | 206 (23.7) | 646 (35.7) | 767 (47.4) | 236 (52.6) | <0.001 |
| Diabetes | | 497 (10.5) | 17 (2.0) | 124 (6.8) | 231 (14.3) | 125 (27.8) | <0.001 |
| Baseline mental health conditions (%) | |  |  |  |  |  |  |
| History of diagnosed mental disorders | | 391 (8.2) | 75 (8.6) | 140 (7.7) | 132 (8.2) | 44 (9.8) | 0.35 |
| Symptoms of depression (CES-D ≥4) | | 529 (11.1) | 105 (12.1) | 189 (10.4) | 169 (10.4) | 66 (14.7) | 0.04 |
| Posterior estimated probability of membership, mean (SD) | | NA | 0.94 (0.12) | 0.91 (0.13) | 0.92 (0.12) | 0.94 (0.12) | - |

Abbreviations: BMI, body mass index (calculated as weight in kilograms divided by height in square meters); CES-D, Center for Epidemiologic Studies Depression Scale; SD, standard deviation. WC, waist circumference.

^†^Data are mean (SD) or n (%) for continuous and categorical variables, as appropriate. The low-stable WC pattern, ranged from 76 to 79 cm (healthy WC); moderate-stable pattern, range of 88 to 92 cm; elevated-stable pattern, range of 100 to 105 cm; high-increasing pattern, range of 115 to 122 cm.

^#^Data are missing for ethnicity for 123 participants (2.6%), marriage for 134 (2.8%), education for 140 (2.9%), smoking for 123 (2.6%), sleep duration for 235 (4.9%), and 123 for comorbidity (2.6%).

**Table S2. Association of genetic and composite lifestyle factors with obesity trajectory compared with the low-stable pattern**

|  | **No (%)** | **ORs (95% CI)** ^†^ (p value) | | | | |
| --- | --- | --- | --- | --- | --- | --- |
|  |  | **Genetic factors** | | | **Lifestyle factors**^*^ | |
| **General obesity, BMI trajectory**^#^ |  |  | | |  | |
| Low-stable | 1175 (24.7%) | 1 [Reference] | | | 1 [Reference] | |
| Moderate-stable | 2013 (42.4%) | 1.96 (1.51-2.56) | | <0.001 | 0.97 (0.82-1.14) | 0.74 |
| Moderate-increasing | 375 (7.9%) | 2.88 (2.00-4.14) | | <0.001 | 1.14 (0.88-1.48) | 0.28 |
| High-decreasing | 235 (4.9%) | 3.52 (2.30-5.34) | | <0.001 | 1.00 (0.72-1.37) | 0.87 |
| High-stable | 953 (20.1%) | 4.31 (3.28-5.73) | | <0.001 | 1.30 (1.07-1.57) | <0.01 |
| **Central obesity, WC trajectory**^#^ |  |  | | |  | |
| Low-stable | 871 (18.3%) | 1 [Reference] | | | 1 [Reference] | |
| Moderate-stable | 1812 (38.1%) | 1.72 (1.30-2.28) | <0.001 | | 1.18 (0.97-1.42) | 0.10 |
| Elevated-stable | 1619 (34.1%) | 2.50 (1.88-3.35) | <0.001 | | 1.29 (1.05-1.58) | 0.01 |
| High-increasing | 449 (9.5%) | 3.91 (2.75-5.58) | <0.001 | | 1.80 (1.37-2.34) | <0.001 |

Abbreviation: BMI, body mass index; WC, waist circumference; OR, odds ratio.

^†^Models were adjusted for age, sex, ethnicity, marital status, employment, and education.

^*^A composite healthy lifestyle score was calculated based on 4 modifiable healthy lifestyle factors including smoking status, alcohol consumption, physical activity, and sleep duration. Individuals’ scores were summed to create an unweighted score, and then classed as favorable (score 0–1) and unfavorable (score 2–4) lifestyle category.

^#^During 1998 to 2012, five distinct BMI trajectories were identified including the low-stable BMI pattern ranged from 22.8 to 22.9 kg/m² (stable healthy body weight); moderate-stable pattern, range of 26.6 to 27.4 kg/m² (stable overweight); moderate-increasing pattern, mean increase from 28.4 to 32.5 kg/m² (overweight to obese); high-decreasing pattern, mean decrease from 31.5 to 28.6 kg/m² (obese to overweight); and high-stable pattern, range of 34.3 to 35.4 kg/m² (stable obese). Four distinct WC trajectories including the low-stable WC pattern, ranged from 76 to 79 cm (healthy WC); moderate-stable pattern, range of 88 to 92 cm; elevated-stable pattern, range of 100 to 105 cm; high-increasing pattern, range of 115 to 122 cm.

**Table S3. Subgroup analyses on the association of BMI trajectory with incident dementia**

|  |  | **BMI trajectory**^†^ | | | | | ***P* value for interaction** |
| --- | --- | --- | --- | --- | --- | --- | --- |
| **Outcome**^#^ | | **Low-stable** | **Moderate-stable** | **Moderate-increasing** | **High-decreasing** | **High-stable** |  |
| **Age, y** |  |  |  |  |  |  |  |
| <65 (n=1173) |  |  |  |  |  |  | 0.30 |
| Multivariable-adjusted HR (95% CI)^#^ | | 1 [Reference] | 1.35 (0.73-2.50) | 1.91 (0.75-4.91) | 2.05 (0.81-5.18) | 1.04 (0.40-2.70) |  |
| ≥65 (n=3455) | |  |  |  |  |  |  |
| Multivariable-adjusted HR (95% CI)^#^ | | 1 [Reference] | 1.22 (0.90-1.65) | 1.08 (0.64-1.81) | 1.08 (0.66-1.78) | 1.52 (1.09-2.14) |  |
| **Sex** | |  |  |  |  |  |  |
| Male (n=2044) | |  |  |  |  |  | 0.10 |
| Multivariable-adjusted HR (95% CI)^#^ | | 1 [Reference] | 1.08 (0.69-1.69) | 1.07 (0.50-2.28) | 0.74 (0.31-1.80) | 1.07 (0.61-1.89) |  |
| Female (n=2707) | |  |  |  |  |  |  |
| Multivariable-adjusted HR (95% CI)^#^ | | 1 [Reference] | 1.33 (0.95-1.87) | 1.34 (0.77-2.36) | 1.50 (0.90-2.48) | 1.82 (1.25-2.66) |  |
| **Hypertension** | |  |  |  |  |  |  |
| Yes (n=1855) | |  |  |  |  |  | 0.78 |
| Multivariable-adjusted HR (95% CI)^#^ | | 1 [Reference] | 1.03 (0.65-1.62) | 1.17 (0.61-2.22) | 1.13 (0.59-2.18) | 1.44 (0.88-2.36) |  |
| No (n=2896) | |  |  |  |  |  |  |
| Multivariable-adjusted HR (95% CI)^#^ | | 1 [Reference] | 1.46 (1.04-2.05) | 1.30 (0.67-2.50) | 1.38 (0.76-2.50) | 1.63 (1.06-2.50) |  |
| **Physical activity** | |  |  |  |  |  |  |
| Moderate/vigorous activity ≥1/w (n=3404) | |  |  |  |  |  | 0.91 |
| Multivariable-adjusted HR (95% CI)^#^ | | 1 [Reference] | 1.28 (0.85-1.94) | 1.12 (0.51-2.43) | 1.16 (0.55-2.44) | 1.44 (0.86-2.42) |  |
| Moderate/vigorous activity <1/w (n=1224) | |  |  |  |  |  |  |
| Multivariable-adjusted HR (95% CI)^#^ | | 1 [Reference] | 1.16 (0.81-1.65) | 1.05 (0.60-1.84) | 1.14 (0.66-1.97) | 1.18 (0.79-1.76) |  |

Abbreviation: BMI, body mass index; HR, hazard ratio.

^†^During 1998 to 2012, five distinct BMI trajectories were identified including the low-stable BMI pattern ranged from 22.8 to 22.9 kg/m² (stable healthy body weight); moderate-stable pattern, range of 26.6 to 27.4 kg/m² (stable overweight); moderate-increasing pattern, mean increase from 28.4 to 32.5 kg/m² (overweight to obese); high-decreasing pattern, mean decrease from 31.5 to 28.6 kg/m² (obese to overweight); and high-stable pattern, range of 34.3 to 35.4 kg/m² (stable obese). Four distinct WC trajectories including the low-stable WC pattern, ranged from 76 to 79 cm (healthy WC); moderate-stable pattern, range of 88 to 92 cm; elevated-stable pattern, range of 100 to 105 cm; high-increasing pattern, range of 115 to 122 cm.

^#^Model were adjusted for age, sex, ethnicity, marital status, employment, education, comorbidities, lifestyle factors, and mental health conditions where appropriate.

**Table S4. Subgroup analyses on the association of WC trajectory with incident dementia**

|  |  | **WC trajectory**^†^ | |  | |  | |  |  | | ***P* value for interaction** |
| --- | --- | --- | --- | --- | --- | --- | --- | --- | --- | --- | --- |
| **Outcome**^#^ | | **Low-stable** | **Moderate-stable** | | **Elevated-stable** | | **High-increasing** | | |  |  |
| **Age, y** |  |  |  | |  | |  | | |  |  |
| <65 (n=1173) |  |  |  | |  | |  | | |  | 0.16 |
| Multivariable-adjusted HR (95% CI)^#^ | | 1 [Reference] | 1.32 (0.93-1.86) | | 1.35 (0.94-1.96) | | 1.51 (0.93-2.45) | | |  |  |
| ≥65 (n=3455) | |  |  | |  | |  | | |  |  |
| Multivariable-adjusted HR (95% CI)^#^ | | 1 [Reference] | 2.01 (0.96-4.60) | | 2.12 (0.89-5.04) | | 0.57 (0.07-4.78) | | |  |  |
| **Sex** | |  |  | |  | |  | | |  |  |
| Male (n=2044) | |  |  | |  | |  | | |  | 0.23 |
| Multivariable-adjusted HR (95% CI)^#^ | | 1 [Reference] | 1.14 (0.45-2.90) | | 0.95 (0.38-2.38) | | 1.05 (0.38-2.89) | | |  |  |
| Female (n=2707) | |  |  | |  | |  | | |  |  |
| Multivariable-adjusted HR (95% CI)^#^ | | 1 [Reference] | 1.42 (1.01-1.99) | | 1.70 (1.17-2.46) | | 1.66 (0.89-3.10) | | |  |  |
| **Hypertension** | |  |  | |  | |  | | |  |  |
| Yes (n=1855) | |  |  | |  | |  | | |  | 0.43 |
| Multivariable-adjusted HR (95% CI)^#^ | | 1 [Reference] | 1.73 (0.96-3.12) | | 1.90 (1.05-3.46) | | 1.67 (0.78-3.60) | | |  |  |
| No (n=2896) | |  |  | |  | |  | | |  |  |
| Multivariable-adjusted HR (95% CI)^#^ | | 1 [Reference] | 1.36 (0.92-1.99) | | 1.37 (0.88-2.12) | | 1.63 (0.88-3.03) | | |  |  |
| **Physical activity** | |  |  | |  | |  | | |  |  |
| Moderate/vigorous activity ≥1/w (n=3404) | |  |  | |  | |  | | |  | 0.71 |
| Multivariable-adjusted HR (95% CI)^#^ | | 1 [Reference] | 1.33 (0.81-2.20) | | 1.34 (0.78-2.31) | | 1.07 (0.47-2.43) | | |  |  |
| Moderate/vigorous activity <1/w (n=1224) | |  |  | |  | |  | | |  |  |
| Multivariable-adjusted HR (95% CI)^#^ | | 1 [Reference] | 1.27 (0.84-1.92) | | 1.18 (0.76-1.82) | | 1.17 (0.66-2.09) | | |  |  |

Abbreviation: WC, waist circumference; HR, hazard ratio.

^#†^During 1998 to 2012, four distinct WC trajectories were identified including the low-stable WC pattern, ranged from 76 to 79 cm (healthy WC); moderate-stable pattern, range of 88 to 92 cm; elevated-stable pattern, range of 100 to 105 cm; high-increasing pattern, range of 115 to 122 cm.

^#^Model were adjusted for age, sex, ethnicity, marital status, employment, education, comorbidities, lifestyle factors, and mental health conditions where appropriate.

**Table S5. Sensitivity analyses on the association of BMI trajectories with risk of dementia**

| **BMI trajectory**^†^ | | | | |
| --- | --- | --- | --- | --- |
| **Low-stable** | **Moderate-stable** | **Moderate-increasing** | **High-decreasing** | **High-stable** |
| **SA1:** Exclusion of participants with incident dementia at the first two years of follow-up (n=4591) | | | | |
| 1 [Reference] | 1.36 (0.93-1.98) | 1.51 (0.85-2.71) | 0.85 (0.41-1.76) | 1.90 (1.25-2.88) |
|  | 0.12 | 0.18 | 0.61 | 0.003 |
| **SA2:** Further adjusted for WC at baseline (wave 6, 2012) based on fully adjusted model | | | | |
| 1 [Reference] | 1.47 (1.03-2.12) | 1.44 (0.76-2.72) | 1.51 (0.87-2.63) | 2.04 (1.20-3.47) |
|  | 0.04 | 0.28 | 0.21 | 0.007 |
| **SA3:** Further adjusted for single measure of BMI (wave 6, 2012) based on fully adjusted model | | | | |
| 1 [Reference] | 1.75 (1.16-2.64) | 2.40 (1.15-4.98) | 1.78 (0.98-3.24) | 2.68 (1.30-5.49) |
|  | 0.008 | 0.02 | 0.09 | 0.005 |
| **SA4**: Further adjusted for genetic risk factor associated with dementia | | | | |
| 1 [Reference] | 1.19 (0.87-1.64) | 0.95 (0.53-1.69) | 1.34 (0.81-2.20) | 1.43 (1.00-2.09) |
|  | 0.18 | 0.89 | 0.25 | 0.05 |
| **SA5**: Exclusion of participants with cancer at baseline (n=4464) | | | | |
| 1 [Reference] | 1.22 (0.93-1.60) | 1.29 (0.82-2.03) | 1.17 (0.76-1.82) | 1.52 (1.11-2.08) |
|  | 0.14 | 0.26 | 0.47 | 0.009 |

Abbreviation: BMI, body mass index; WC, waist circumference. HR (95% CI) and p-value are showed.

^†^During 1998 to 2012, five distinct BMI trajectories were identified including the low-stable BMI pattern ranged from 22.9 to 22.8 kg/m² (stable healthy body weight); moderate-stable pattern, range of 26.7 to 27.4 kg/m² (stable overweight); moderate-increasing pattern, mean increase from 28.5 to 32.3 kg/m² (overweight to obese); high-decreasing pattern, mean decrease from 31.5 to 29.0 kg/m² (obese to overweight); and high-stable pattern, range of 34.3 to 35.4 kg/m² (stable obese). Four distinct WC trajectories including the low-stable WC pattern, ranged from 75 to 79 cm (healthy WC); moderate-stable pattern, range of 87 to 92 cm; elevated-stable pattern, range of 98 to 104 cm; high-increasing pattern, range of 114 to 121 cm.

^#^Model were adjusted for age, sex, ethnicity, marital status, employment, education, comorbidities, lifestyle factors, and mental health conditions.

**Table S6. Sensitivity analyses on the association of WC trajectories with risk of dementia**

| **WC trajectory**^†^ | | | | |
| --- | --- | --- | --- | --- |
| **Low-stable** | **Moderate-stable** | **Moderate-increasing** | **High-decreasing** |  |
| **SA1:** Exclusion of participants with incident dementia at the first two years of follow-up (n=4591) | | | | |
| 1 [Reference] | 1.41 (0.92-2.18) | 1.71 (1.09-2.69) | 1.73 (0.93-3.21) |  |
|  | 0.13 | 0.02 | 0.10 |  |
| **SA2:** Further adjusted for BMI at baseline (wave 6, 2012) based on fully adjusted model | | | | |
| 1 [Reference] | 1.29 (0.85-1.96) | 1.25 (0.72-2.17) | 0.99 (0.40-2.41) |  |
|  | 0.33 | 0.52 | 0.92 |  |
| **SA3:** Further adjusted for single measure of WC (wave 6, 2012) based on fully adjusted model | | | | |
| 1 [Reference] | 1.45 (0.93-2.26) | 1.52 (0.80-2.89) | 1.75 (0.65-4.73) |  |
|  | 0.11 | 0.19 | 0.23 |  |
| **SA4:** Further adjusted for genetic risk factor associated with dementia | | | | |
| 1 [Reference] | 1.51 (1.02-2.22) | 1.66 (1.10-2.50) | 1.23 (0.67-2.29) |  |
|  | 0.007 | 0.007 | 0.22 |  |
| **SA5**: Exclusion of participants with cancer at baseline (n=4464) | | | | |
| 1 [Reference] | 1.45 (1.06-2.00) | 1.50 (1.07-2.10) | 1.73 (1.08-2.77) |  |
|  | 0.02 | 0.01 | 0.02 |  |

Abbreviation: BMI, body mass index; WC, waist circumference. HR (95% CI) and p-value are showed.

^†^During 1998 to 2012, four distinct WC trajectories were identified including the low-stable WC pattern, ranged from 76 to 79 cm (healthy WC); moderate-stable pattern, range of 88 to 92 cm; elevated-stable pattern, range of 100 to 105 cm; high-increasing pattern, range of 115 to 122 cm.

^#^Model were adjusted for age, sex, ethnicity, marital status, employment, education, comorbidities, lifestyle factors, and mental health conditions.
